# Amniotic fluid-derived mesenchymal stem cells as a therapeutic tool against cytokine storm: a comparison with umbilical cord counterparts

**DOI:** 10.1186/s13287-025-04262-0

**Published:** 2025-03-28

**Authors:** Salvatore Vaiasicca, David W. James, Gianmarco Melone, Omar Saeed, Lewis W. Francis, Bruna Corradetti

**Affiliations:** 1 Advanced Technology Center for Aging Research, IRCCS INRCA, Ancona, Italy; 2 Department of Life and Environmental Life, Polytechnic University of Marche, Ancona, Italy; 3 Centre of NanoHealth, Swansea University Medical School, Swansea, UK; 4 Center for Precision Environmental Health, Baylor College of Medicine, Houston, TX USA; 5 Department of Medicine, Section Oncology/Hematology, Baylor College of Medicine, Houston, TX USA

**Keywords:** Mesenchymal stem cells, Amniotic fluid, Umbilical cord, Cytokine storm, Transcriptomic analysis, Regulatory moieties, Immunosuppression, GSEA, COVID-19

## Abstract

**Background:**

Several immunosuppressive therapies have been proposed as key treatment options for critically ill patients since the first appearance of severe acute respiratory syndrome coronavirus 2. Mesenchymal stem cells (MSCs) from different sources have been considered for their potential to attenuate the cytokine storm associated to COVID-19 and the consequent multi-organ failure, providing evidence for safe and efficacious treatments. Among them, administration of umbilical cord-derived MSCs (UC-MSCs) has demonstrated a significant increase in survival rates, largely due to their potent immunosuppressive properties.

**Methods:**

We applied next-generation sequencing (NGS) analysis to compare the transcriptomic profiles of MSCs isolated from two gestational sources: amniotic fluid (AF) obtained during prenatal diagnosis and their clinically relevant umbilical cord counterparts, for which datasets were publicly available. A full meta-analysis was performed to identify suitable GEO and NGS datasets for comparison between AF- and UC-MSC samples.

**Results:**

Transcriptome analysis revelaed significant differences between groups, despite both cell lines being strongly involved in the tissue development, crucial to achieve the complex task of wound healing. Significantly enriched hallmark genes suggest AF-MSC superior immunomodulatory features against signaling pathways actively involved in the cytokine storm (i.e., IL-2/STAT, TNF-a/NFkB, IL-2/STAT5, PI3K/AKT/mTOR).

**Conclusions:**

The data presented here suggest that AF-MSCs hold significant promise for treating not only COVID-19-associated cytokine storms but also a variety of other inflammatory syndromes (i.e., those induced by bacterial infections, autoimmune disorders, and therapeutic interventions). Realizing the full potential of AF-MSCs as a comprehensive therapeutic approach in inflammatory disease management will require more extensive clinical trials and in-depth mechanistic studies.

**Supplementary Information:**

The online version contains supplementary material available at 10.1186/s13287-025-04262-0.

## Introduction

Chronic diseases (including cardiovascular disease, diabetes, and cancer) are the major causes of death and disability worldwide, driving $3.8 trillion in annual health care costs in the US. The pandemic induced by severe acute respiratory syndrome coronavirus 2 (SARS-CoV-2) infection is a heavy contributor to these statistics, in part due to the chronic inflammation associated to viral infection, also known as cytokine storm [1]. The cytokine storm is a condition of uncontrolled systemic hyper-inflammation caused by an excessive cytokine release, leading to multi-organ failure [2, 3] and, in some cases, death. While it is most commonly associated with severe viral infections [4, 5], as in the case of SARS-CoV-2, cytokine storms can also be triggered by a variety of other conditions. These include bacterial infections (i.e., sepsis) [6], autoimmune disorders (i.e., macrophage activation syndrome [7] or hemophagocytic lymphohistiocytosis [8]. Furthermore, the use of potent immunotherapies, including monoclonal antibodies and CAR T-cell treatments, has been also associated with a phenomenon known as cytokine release syndrome (CRS) [9, 10], which shares many similarities with natural cytokine storms. As an example, circulating cells and pro-inflammatory molecules resulting from an acute SARS-CoV-2 infection are responsible for chronic COVID-19 associated endotheliopathy [11, 12], a condition that arises from the persistent impairment of endothelial cell identity exacerbating the inflammatory and pro-thrombotic state, altering vascular permeability [13]. As a result, a second pandemic of degenerative cardiovascular disease (CVD) was predicted as the consequence of the first pandemic of COVID-19. Despite a declined trend in the overall mortality CVD observed in 2022–2023, persistence of excess deaths has been determined among the elderly and different racial/ethnic groups recently, concerning about long-term impacts of COVID-19 on these patient populations [14]. With both cytokines, and inflammation playing a dominant role in the development of COVID-19-induced damage, several immunological therapies, with the potential to attenuate the cytokine storm, have been proposed as key treatment options for critically ill patients [15, 16]. Among them, traditional anti-inflammatory drugs (including corticosteroids and colchicine) [17] and the use of low-dose radiation therapy (e.g., steroids) have showed modest efficacy, while the effectiveness of cytokine blockades (i.e., interleukins) has been only demonstrated when a specific (and targeted) cytokine is known to play role in the disease [18]. Despite significant progress since the initial emergence of COVID-19, side effects of SARS-CoV-2 infection remain unacceptably high and warrants an urgent need to identify a highly innovative therapeutic strategy to rapidly attenuate the cytokine storm.

Naturally occurring MSCs with immunoregulatory, regenerative, and anti-inflammatory properties [19–21] represent a magic bullet for the development of treatment strategies able to reduce symptoms and alleviate any off-target effects induced by microbial infections, including, but not limited to, COVID-19 [19, 21–23]. By virtue of their powerful immunomodulatory ability and their natural ability to migrate to sites of injury, mesenchymal stem cells (MSCs) offer a promising innovative strategy for attenuating the cytokine storm and ultimately improving patients’ outcomes [24]. Preclinical studies have provided robust evidence for their use in treating lung injury and acute respiratory distress syndrome [25, 26]. MSCs can reduce pro-inflammatory cytokines and increase anti-inflammatory factors, ultimately restoring microenvironment homeostasis in pulmonary epithelial and endothelial cells and promoting alveolar fluid clearance [27]. More than 1,000 clinical trials have been conducted worldwide to evaluate the therapeutic potential of MSC-based approaches for tissue repair following chronic deterioration, providing evidence for safe and efficacious treatments (clinicaltrials.gov). They include the 85 ongoing trials for the treatment of the cytokine storm caused by SARS-CoV-2 infections, Placental tissues (i.e., umbilical cord, chorionic villi, amniotic fluid, and membranes) are currently the most used sources of MSCs because they have greater immunosuppressive potential than adult counterparts (i.e., bone marrow and adipose tissue) [22, 28, 29]. MSCs derived from gestational tissues offer advantages over other sources, such as being non-invasive, ethical, and benefiting from high yield collection procedures [30–32]. The two most investigated and used sources of MSCs to challenge SARS-CoV-2 inflammation and its associated consequences include adipose tissue and the umbilical cord, followed by the bone marrow and dental pulp [33, 34]. Many of these studies are aimed at evaluating the safety, efficacy, and therapeutic effect of MSCs, obtained from the previously mentioned allogenic and autologous sources. Umbilical cord mesenchymal stem cells (UC-MSCs) have been widely investigated for their capability to counteract the chronic effects induced by COVID-19 infections. Lanzoni et al. have determined the safety and proved the efficacy of UC-MSC infusions in subjects with acute respiratory distress syndrome associated to COVID-19 [22]. Through a double blind, phase 1/2a, randomized and controlled trial the authors have demonstrated a significant reduction in the inflammatory cytokines in UC-MSC-treated subjects as soon as day 6. Another study has demonstrated UC-MSC potential to influence pro-inflammatory T-helper 2 (Th2) cell shift to an anti-inflammatory phenotype and used UC-MSC as adjuvant therapy in critically ill patients with COVID-19 [35]. Data demonstrated UC-MSC administration increases survival rates by 4.5 times compared with controls, which is mediated by the immunosuppressive features of these cells [35]. MSCs from other gestational sources, including the amniotic membranes and fluid, have been also considered as a new hope for therapies against the multiple proinflammatory cytokine storm in severe patients [20]. Our group has extensive experience in the isolation in vitro testing and in vivo application of MSCs from gestational tissues [36–38], with particular emphasis on amniotic membranes and amniotic fluid (AF-MSCs) and their derivatives [38–41]. In this study, we aim to identify molecular signatures that could mitigate viral infection-induced inflammation in patient-derived AF-MSCs. We performed a comprehensive molecular characterization of AF-MSCs, focusing on their immunosuppressive and immunomodulatory properties. To further explore their therapeutic potential, we conducted Next-Generation Sequencing (NGS) analysis of the AF-MSC transcriptome. The resulting datasets were compared with publicly available RNA transcriptomes of UC-MSCs, which have been extensively studied for their effective immunomodulatory roles. This comparison was designed to identify relevant molecular signatures with potential efficacy in managing SARS-CoV-2-associated cytokine storms. To ensure robust and accurate results, both datasets underwent rigorous quality control (QC) analysis.

## Materials and methods

### Amniotic fluid harvesting and mesenchymal stem cell (MSC) culture

Amniotic fluid samples were obtained from healthy pregnant women undergoing prenatal diagnosis at the Cytogenetic Laboratory Children’s Hospital Salesi (Ancona, Italy) upon informed written consent for their use for research purposes. The study was approved by the Regional Institutional Review Board (Comitato Etico Regione Marche, Italy) and was conducted in accordance with the Declaration of Helsinki. AF-MSCs were maintained in stem cell standard culture medium constituted by High Glucose-Dulbecco’s Modified Eagle Medium (HG-DMEM) (ThermoFisher Scientific) supplemented with 10% fetal bovine serum (FBS) (Sigma-Aldrich), 1% L-glutamine and 100 U/ml Penicillin/Streptomycin (P/S) solution (Sigma-Aldrich). The human leukemia monocytic cells (ThP-1), purchased from ATCC, were cultured in Roswell Park Memorial Institute (RPMI-1640) media (Gibco) supplemented with 10% FBS (Corning), 1% L-glutamine, and 2% P/S. For culture maintenance, cells were plated at a cell density of 5 × 10^3^ cells/cm^2^ and maintained in a humidified incubator at 37 °C with 5% CO_2_.

### Proliferation assays

*Growth curves.* Growth curves were evaluated at passage 2 (P2) as previously reported [42, 43]. Briefly, AF-MSCs were plated at density 10^4^ cells/well into 12-well tissue culture polystyrene dishes (EuroClone). Every 2 days over a 10 day period, cells were trypsinized and counted by using trypan blue exclusion dye method and cell number and viability were evaluated. Three biological replicates (*n* = 3) were considered.

*Doubling time (DT)*. AF-derived MSCs were seeded into six-well tissue culture polystyrene dishes at the density of 9.5 × 10^3^ cells/well, trypsinized every 4 days, counted and replated at the same density. DT was assessed from P2 to P5. DT values were obtained for each passage according to the formula DT = CT/CD, where CT represents the culture time and CD = ln(Nf/No)/ln2 represents the number of cell generations (Nf represents the number of cells at confluence, No represents the number of seeded cells) [37, 44].

*Colony-forming unit-fibroblastic (CFU-F) assay.* AF derived MSCs (at P2) were seeded at different densities (namely, 1.5 × 10^3^, 3 × 10^3^, 4.5 × 10^3^ cells/cm^2^) in six-well plates for 2 weeks in stem cell standard culture medium, as previously reported [45]. At the end of the 2 weeks culture period, colonies were fixed with 1% paraformaldehyde (PFA), stained with Giemsa at room temperature (RT) and washed twice. Colonies formed by 15–20 nucleated were counted with an inverted microscope (Meiji Techno).

### Morphological assessment and molecular characterization

*AF-MSCs morphology.* AF-MSC morphology was visualized by fluorescence microscopy (Olympus BX51), equipped with the Spot Advanced software. To specifically highlight cell cytoskeleton ActinGreen (Life Technologies) was used. Nuclei was counterstained with Hoechst (Sigma-Aldrich). Briefly, cells were seeded at the density of 14 × 10^3^/well in 8-well chamber slide and let adhere overnight. Cells were subsequently washed twice in PBS, fixed with 4% PFA for 15 min and permeabilized with 0.1% Triton (in PBS) for 10–15 min. MSCs were then blocked in 1% bovine serum albumin (BSA) in PBS for 30 min at room temperature and incubated with ActinGreen for 20 min at RT in the dark. Incubation with Hoechst (1 μg/ml) was performed 5 min before visualization occurred. Slides were kept in the dark until observation started.

*Flow cytometry.* AF-MSCs were characterized for the expression of MSC- associated markers at P2. Cells were trypsinized, washed with PBS and fixed with 75% ethanol. They were subsequently incubated for 20 min in 0.5% BSA in PBS to block non-specific sites. Samples were stained with directly conjugated antibodies as follows: phycoerythrin (PE)-conjugated ecto-5’-nucleotidase (PE-CD73; BioLegend), Allophycocyanin (APC)-conjugated thymocyte differentiation antigen 1 (APC-CD90; BioLegend) and Integrin b1 (APC-CD29; BioLegend), and fluorescein isothiocyanate (FITC)-conjugated glycoprotein CD44 (FITC-CD44; BioLegend). Incubation was performed for 45 min at room temperature in the dark. The excess of antibody was removed by washing cells twice with PBS. Analyses were carried out using Guava Easycyte Millipore flow cytometer with GuavaSoft 2.2.3.

*Gene expression analysis.* Gene expression analysis was performed on AF-MSCs to determine the expression of pluripotent (*Nanog* and *Oct-4*)-, mesenchymal (*Cd105*, C*d90*, *Cd44*, and *Cd73*)-, hematopoietic (*Cd45* and *Cd34*)-associated markers. Total RNA was extracted from cells using TRI Reagent® (Sigma-Aldrich) according to the manufacturer’s instructions. RNA concentration and purity were measured by Nanodrop® Spectrophotometer (Nanodrop® ND1000). cDNA was synthesized from 500 ng total RNA using a PrimeScript™ RT-Reagent Kit (Takara). Gene expression was evaluated using human specific oligonucleotide primers (Table 1, [40]). Primers used were designed using open-source Primer-BLAST, across an exon–exon junction to avoid genomic DNA amplification and make manual corrections to make better amplification. Quantitative PCRs were performed with SYBR® green method in a StepOneTM Real-Time PCR System, StepOne cycler software v2.3. Triplicate PCR reactions were carried out for each sample analyzed. The thermal profile for all reactions was 10 min at 95 °C and then 40 cycles of 15 s at 95 °C, 1 min at 60 °C. Fluorescence monitoring occurred at the end of each cycle. Human glyceraldehyde-3-phosphate dehydrogenase (*Gapdh*) was employed as a reference gene in each sample to standardize the results by eliminating variation in cDNA quantity.

**Table 1:** Oligonucleotides used to evaluate the expression of mesenchymal, pluripotent, hematopoietic, and immunosuppressive markers (S = sense primer, A = antisense primer, Tm = melting temperature, bp: base pairs)

GENE	Sequences (5’ → 3’)	Tm (°C)	Product Size (bp)
Mesenchymal markers			
CD44 molecule (*Cd44)*	S: GGAGCAGCACTTCAGGAGGTTAC	63	129
	A: GGAATGTGTCTTGGTCTCTGGTAGC		
5’-nucleotidase, ecto (*Cd73)*	S: GCTCTTCACCAAGGTTCAGC	59	203
	A: GTGGCTCGATCAGTCCTTCC		
Thy-1 cell surface antigen (*Cd90)*	S: CTTTGGCACTGTGGGGGTGC	61	211
	A: GATGCCCTCACACTTGACCAG		
Endoglin (*Cd105)*	S: CCTGGAGTTCCCAACGGGCC	62	186
	A: GGCTCTTGGAAGGTGACCAGG		
Hematopoietic markers			
CD34 molecule (*Cd34)*	S: GTGTCTACTGCTGGTCTTGG	58	200
	A: CAGTGATGCCCAAGACAGC		
CD45 molecule (*Cd45)*	S: GACAACAGTGGAGAAAGGACG	60	170
	A: GCTGTAGTCAATCCAGTGGGG		
Pluripotent markers			
Pou class 5 homeobox 1 (*Oct-4)*	S: CGATCAAGCAGCGACTATGC	60	200
	A: AGAGTGGTGACGGAGACAGG		
Nanog homeobox (*Nanog)*	S: GCAAGAACTCTCCAACATCC	56	178
	A: GGTCTGGTTGCTCCACAT		
Pro- and Anti-inflammatory markers			
Prostaglandin E-receptor 2 (*Pge-2)*	S: GGAAGGAGAAAGCTCGCAAC	58	173
	A: TGAGCCAGTACTTATTGCCG		
Tumor necrosis factor-alpha (*Tnf-α*)	S: TCTGGCCCAGGCAGTCAGATC	64	180
	A: TACAGGCCCTCTGATGGCACC		
Transforming growth factor-beta (*Tgf-b)*	S: TGGTCATGAGCTTCGTCAAC	58	171
	A: TCTCATTGTCGAAGCGTTCC		
Housekeeping gene			
Glyceraldehyde-3-phosphatase dehydrogenase (*Gapdh*)	S: TCCACTGGCGTCTTCACC	68	78
	A: GGCAGAGATGATGACCCTTT		

### Evaluation of AF-MSC immunosuppressive and immunomodulatory potential

*Immunosuppressive potential.* To evaluate the immunosuppressive potential of AF-MSCs cells (at P2) were seeded at the density of 2 × 10^4^/well in 24-well plates and cultured for 24 h at 37 °C. They were subsequently exposed for 24 and 48 h to the pro-inflammatory cytokines, namely human tumor necrosis factor-alpha (TNF-α, 20 ng/ml*)* and interferon-gamma (IFN-γ, 20 ng/ml*)* (Peprotech). The expression of immunosuppressive markers, including *Tnf-*α, prostaglandin E2 (*Pge2*), and tumor growth factor-beta (*Tgf-*β) was evaluated by quantitative PCR using primers (Table 1).

*AF-MSCs and monocytic cell co-cultures.* Functional assays were also set up to determine the influence of AF-MSCs in reducing the proliferation of monocytic cells (the ThP-1 cell line). Specifically, we compared the effect of AF-MSCs by testing three different modalities: (1) AF-MSCs were seeded together with ThP-1 cells (Cell-to-cell contact, 1:10 MSCs:ThP-1 ratio), (2) ThP-1 cells were exposed to the media conditioned by AF-MSCs (Conditioned Media, CM), and (3) AF-MSCs and ThP-1 cells were co-cultured (Transwell system: MSCs vs ThP-1, 1:10 ratio). CM from AF-MSCs was produced by culturing cells for 2 days into T25 flasks at a density of 20 × 10^3^ cells/cm^2^ in Standard Culture Medium supplemented with 10% of exosome-depleted FBS (Gibco), 1% L-glutamine and 100 U/ml P/S solution (Sigma-Aldrich). At the end of the culture period, CM was collected, centrifuged at 500 × g to remove cell debris, and kept at − 80 °C until further analysis. ThP-1 cell proliferation was induced by stimulating them with 2% phytoemagglutinin (PHA; Sigma-Aldrich) in HG-DMEM complete media. ThP-1 in absence of MSCs were used as controls. Monocytes proliferation was assessed after 2 days in culture. To perform co-culture analyses, ThP-1 cells were stained with BD Horizon Violet Cell Proliferation Dye 450 (VPD450; BD Biosciences) for 10 min, washed in PBS and cultured in 24-well plates at the density of 5 × 10^5^/well. 48 h following exposure to AF-MSCs and derivatives, ThP-1 cells were harvested, and their proliferation was assessed by flow cytometry. Hundred thousand events were acquired for each condition using Guava Millipore cytometer and analyzed with FlowJo® software (FlowJo LLC).

*Effect of conditioned media from AF-MSCs and macrophages.* Macrophages (MF) were generated from ThP-1 cells following established protocols. To generate MF, ThP-1 cells were seeded at the density of 5 × 10^5^ in 6 well plate and treated with 100 nM/mL phorbol-12-myristate-13-acetate (PMA, Sigma-Aldrich) for 24 h. Pro-inflammatory macrophages (MF1) were obtained upon incubation of MF with 20 ng/mL TNF-a and 20 ng/mL IFN-γ (ThermoFisher) for 18 h. To determine the role of AF-MSCs in modulating MF and potentially reduce inflammation, undifferentiated MF or MF1were exposed to the media conditioned by AF-MSCs upon priming with pro-inflammatory cytokines for 48 h (iAF-MSCs). Resulting macrophages were processed to evaluate the expression of anti- and pro-inflammatory genes on MF and MF1, respectively. Briefly, total RNA was extracted using Trizol reagent (Invitrogen) and purified to eliminate genomic DNA, protein and organic contaminations using the RNeasy Mini Kit (Qiagen). The concentration and integrity of RNA samples were assessed using the NanoDrop ND-2000 spectrophotometer (NanoDrop Technologies). cDNA synthesis was performed using the RT2 First Strand Kit (Qiagen) according to the manufacturer’s instructions. Transcribed products were analyzed using commercially available master mix (Applied Biosystems) and the appropriate target probes to detect expression of pro-inflammatory (*iNos*: Hs01075529_m1, *Tnf-a*: Hs00174128_m1, *Il-6*: Hs00174131_m1) and anti-inflammatory (*Mrc1*: Hs00267207_m1, *Il10*: Hs00961622_m1, *Ccl22*: Hs01574247_m1, *Tgf-b1*: Hs00998133_m1) markers on an ABI StepOne plus Detection System (Applied Biosystems). Expression levels were normalized to the reference gene (*Gapdh*: Hs02758991_g1).

### RNA-Sequencing analysis by NGS library preparation

*RNA extraction and quality check.* For NGS sequencing, three samples of AF-MSCs at P2 were used. Total RNA was extracted and purified using Total RNA Purification Plus Kit (Norgen, Biotek Corp) and processed by the Functional Genomic Center at the University of Verona (Italy). The concentration and integrity of all RNA samples were assessed using the RNA 6000 Nano Kit (Agilent Technologies). RNA samples showed an integrity number (RIN) > 9.

*RNA-seq library preparation.* RNA-seq library preparation was performed using the TruSeq stranded mRNA kit (Illumina) from 1 μg of RNA samples. Libraries size was assessed by capillary electrophoretic analysis with the Agilent 4200 Tape station. RNA libraries were analyzed on an Illumina NextSeq 500 sequencer using 75nt single reads.

### Identifying and downloading datasets

Suitable UC-MSC RNA-seq datasets were identified by querying the National Center for Biotechnology Information (NCBI) geo repository using a PRISMA approach that involved several search terms. From the repository GSE118808 we obtained 4 transcriptomic datasets obtained from patients-derived UC-MSC samples. Similarly to our samples, we utilized for MSC isolation, the samples chosen for this study were not “primed” by being exposed to SARS-CoV-2, in the form of cytokines storm or the virus itself. Sequence Reads Archive (SRA) files were downloaded to an internal server and converted to Fastq files using the fasterq-dump application available in NCBI. The sequencing quality for each Fastq file (house and downloaded) was assessed using software FastQC (http://www.bioinformatics.babraham.ac.uk/projects/fastqc/).

### Alignment to genome and gene count tables

Prior to alignment, adaptor contaminants were removed using Scythe software (https://github.com/vsbuffalo/scythe). Furthermore, low quality read tails were removed using sickle software (https://github.com/najoshi/sickle). Once done, reads were aligned to Human reference genome GRCh38 (hg38) using STAR aligner (https://www.ncbi.nlm.nih.gov/pmc/articles/PMC3530905/). The quantmode option in STAR was used to create gene count tables for genes. Gene coordinates were obtained from gencode human release 30 (GRCh38.p12) (https://www.gencodegenes.org/human/release_30.html) for accurate mapping. The Mapping statistics for reads in each sample were obtained from STAR. Raw and processed data are deposited in the Gene Expression Omnibus (GEO) database with accession number GSE240855.

### Analysis of differentially gene expression (DEGs) and Pathways enrichment analysis

Once the alignment, QC and mapping statistics were completed, differential expression analysis was conducted through DESeq2 in R [46]. *P*-values of significantly dysregulated genes were assessed using the Wald test within DESeq2. Following differential analysis, raw count data was transformed using VST transform to remove the dependence of the variance on the mean. Custom code was curated from the results of DESeq2 to create a correlation heatmap with associated dendrograms and a principal component analysis (PCA) plot to confirm both sample groups (AF against UC) were unique. Following the confirmation, the top 25 enriched genes were mapped in heatmap and subsequently investigated considering both experimental groups (AF-MSCs vs UC-MSCs and UC-MSCs vs AF-MSCs). DEGs for each sample were selected via specific significance parameters (*p* adjusted value < 0.05 and base-2 log fold change > 2). However, due to the identification of low counts and high dispersion among DEGs, the datasets underwent a log fold shrinkage via ‘apeglm’ allowing for a reduction in noise [47]. This shrinkage estimator uses a heavy-tailed Cauchy prior distribution for effect sizes and therefore has lower bias than other shrinkage estimators. In using ‘apeglm’, the visualization of DEGs among the sample groups became clearer. To visualize the DEGs between UC and AF-MSC MA and Volcano plot were created using the ggplot2 package.

### Pathways enrichment analysis

Pathway enrichment was observed via gene set enrichment analysis (GSEA) (version 4.1.0) software provided by the Molecular Signatures Database with pre-selected parameters set by the software [48, 49]. Leading edge analysis was conducted through the same software. Both investigational analyses utilized hallmarks (version 7.4) as the gene set database for pathway comparison between the sample groups.

#### Statistical analysis

Statistical analysis was performed using GraphPad Instat 3.00 for Windows (GraphPad Software). Three replicates for each experiment (doubling time, colony forming unit, quantitative PCR, cytometry analysis and monocytes proliferation test) were performed and the results are reported as mean ± standard deviation (SD). One-way analysis of variance for multiple comparisons by the Student–Newman–Keuls multiple comparison test were used to assess differences between groups. Differences were considered statistically significant for *p* values < 0.05.

## Results

### AF-MSC proliferation, morphology, and molecular phenotype

**Fig. 1:**
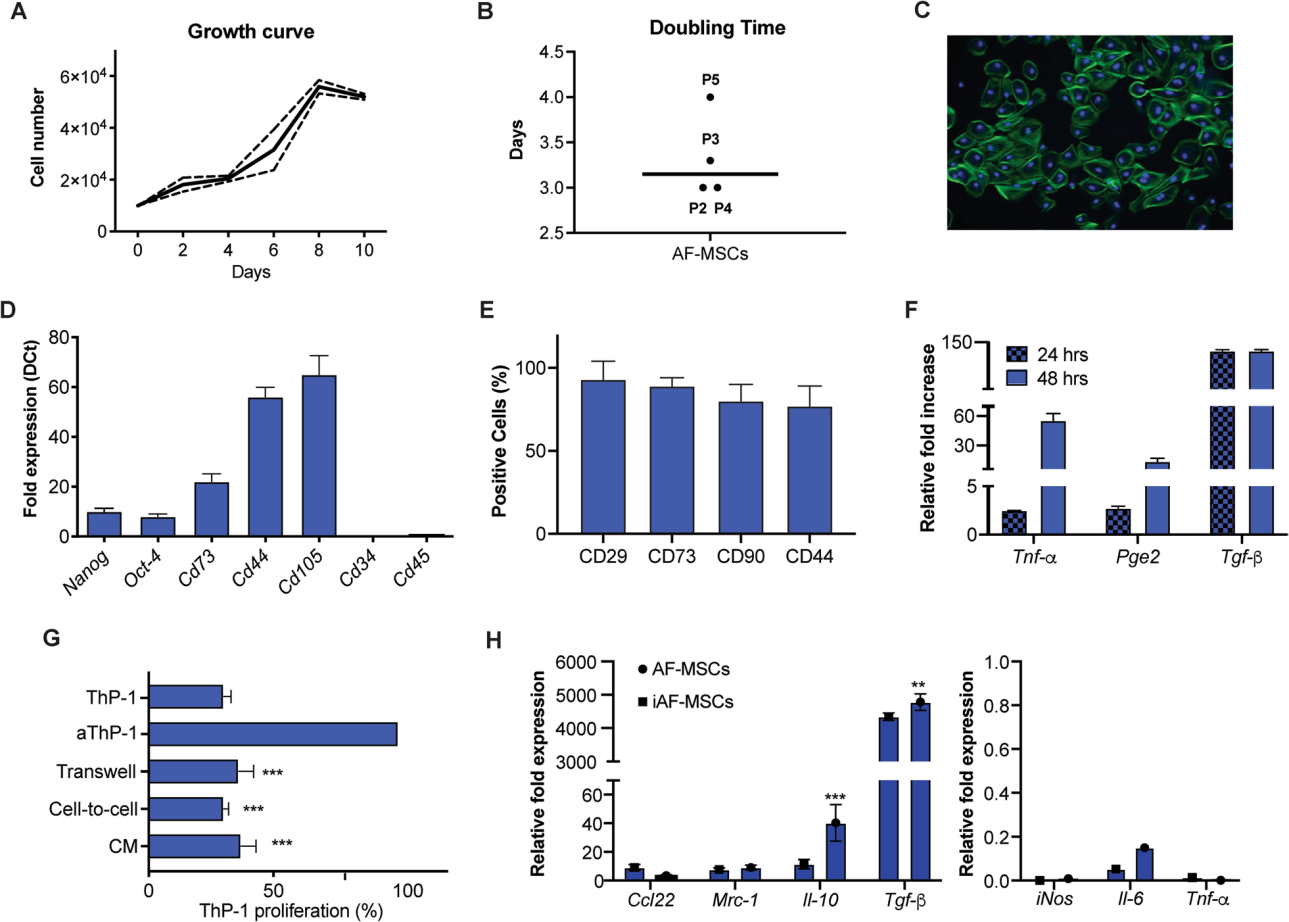
AF-MSC proliferation capacity, morphology, phenotype and immunosuppressive effect. Graph showing the growth of AF-MSCs (at P2) over a 10-day culture period **A**. Doubling time for AF-MSCs at different passages (from P2 to P5) **B**. AF-MSC morphology visualized by fluorescence microscopy for the identification of phalloidin (green) and the cell nucleus are stained using DAPI (blue) **C**. Images taken at 20 × magnification, scale bar: 10 µm. Quantitative PCR analysis for the expression of pluripotent (*Nanog, Oct4*) mesenchymal (*cd73, cd90, cd44 and cd105*) and hematopoietic (*cd34* and *cd45*) markers in AF-MSCs at passage P2 **D**. Flow cytometric analysis revealing the percentage of AF-MSCs positive for MSC-associated markers (CD29, CD73, CD90 and CD44). Results are presented as percentage of marker-positive cells and as average of three biological replicates ± standard deviation **E**. Expression of immunosuppressive markers in AF-MSCs as revealed by qPCR following 24 and 48 h treatment with a cocktail of pro-inflammatory cytokines (TNF-α and IFN-g, 20 ng/ml) **F**. Data represent the average of fold as fold-change compared with the expression levels found in the untreated cells (* = *p* > 0.05; ** = *p* < 0.01). Immunosuppressive effect of AF-MSCs on monocyte proliferation (ThP-1 cell line) as shown by flow cytometry **G**. Three different culture conditions were set: 1) Cell-to-cell contact, 2) Conditioned Media (CM), and 3) Transwell system. Resting and PHA-activated ThP-1 cells (aThP-1) were included in the study as negative and positive controls, respectively. Data represent the percentage ± SD of three biological replicates (* = *p* > 0.05; ** = *p* < 0.01; *** = *p* < 0.001). **H** qPCR analysis of anti(*Il-10, Tgf-b, Ccl22, Mrc1*)- and pro(*iNos*, *IL-6*, *Tnf-a*)- inflammatory gene expression in undifferentiated macrophages (MF) or inflamed macrophages (MF1), respectively, exposed to CM from AF-MSC grown in standard conditions (AF-MSCs) or primed with pro-inflammatory cytokines (iAF-MSCs). Data are normalized to housekeeping gene expression (*Gapdh*) and presented as fold change relative to MF not exposed to CM for anti-inflammatory genes and to MF1 for pro-inflammatory markers (*n* = 3). Error bars represent the mean ± SD; ** = *p* > 0.05; ** = *p* < 0.01)

AF-MSCs were cultured and growth curves analysed based on cell proliferation, at P2 AF-MSCs demonstrated a growth curve with an initial lag phase of 2 days that decreased after 8 days (Fig. 1A). Doubling time values showed an average of 3.23 ± 0.47, with a similar trend from P2 to P4 and a slight increase observed at P5 (*p* < *0.05*) (Fig. 1B). AF-MSCs display a clonogenic capacity, with a direct correlation between the increase of CFU frequency and the increase of cell seeding density (Table 2). As shown in Fig. 1C, AF-MSCs display a cobblestone-like morphology. The evaluation of mesenchymal and pluripotent markers by quantitative PCR revealed AF-MSC share embryonic (*Nanog* and *Oct-4*) and adult (*Cd73*, *Cd90, Cd44, Cd105*) features. No contamination in terms of hematopoietic cells was observed as demonstrated by the lack of expression for *Cd34* and *Cd45* (Fig. 1D). Immunophenotypic analysis was performed to examine the expression of the main mesenchymal markers, with strong positive signal for MSC-specific surface markers such as CD29, CD73, CD90, and CD44. Histograms and expression profiles of these cells at passage 4 are shown in Supplementary Fig. 1 and Fig. 1E, respectively.

**Table 2:** Colony forming unit (CFU) – fibroblastic like capability of AF-MSCs at different seeding density (cells/cm^2^). Data are reported as the average of three biological replicates ± standard deviation)

Density	Total cells	CFU	1 CFU each
1500	14,250	2.5 ± 0.7	7125
3000	28,500	4 ± 0.4	5700
4500	42,750	10.5 ± 0.7	4275

### AF-MSC immunosuppressive and immunomodulatory potential

The exposure to the inflammatory environment generated by the presence of TNF-a and IFN-g resulted in increased expression of inflammatory genes compared to unexposed cells (Fig. 1F). Following twenty-four hours, a slight increase was found in the mRNA levels of *Tnf-*a and *Pge2,* with values at 1.50 ± 0.01–1.72 ± 0.22-fold compared to control (*p* < 0.01). The levels of the *Tgf-b* expression showed a significant 145 ± 0.4-fold increase (*p* < 0.01). After 48 h a further 57.4 ± 3.2-, 12.1 ± 1.3-fold increase was observed compared to control for *Tnf-α* and *Pge2* respectively (*p* < 0.01). The level of *Tgf-β* expression did not change compared to 24 h, remaining high. The immunosuppressive potential was functionally evaluated through ThP-1 proliferation, a downregulation of PHA-stimulated ThP-1 proliferation was observed, under all test conditions (cell-to-cell contact, transwell cultures and using CM), following 2 days of co-culture with AF-MSCs (Fig. 1G). The inhibitory effect observed in AF-MSCs was greater following direct contact with ThP-1, showing a significant 70% decrease in ThP-1 proliferation (*p* < 0.05). A 63% reduction was observed when monocytic cells were exposed to the paracrine signals released by AF-MSCs (CM) and a 64% reduction was found when the two cell populations co-existed in a transwell system. To assess their immunomodulatory effect, undifferentiated or inflamed macrophages (MF and MF1, respectively) were exposed for 48 h to CM obtained from AF-MSCs grown in standard conditions or in presence of pro-inflammatory cytokines (iAF-MSCs). Gene expression analysis revealed an overall increase in the expression of markers associated to anti-inflammatory MF (MF2) in both groups, with a significant upregulation occurring upon exposure to AF-MSCs for *Il-10* and Tgf-b (Fig. 1H, p < *0.001*). The presence of CM from AF-MSCs and iAF-MSCs determined a marked refuction in the expression of pro-inflammatory markers known to be present in MF1.

### RNA-Seq analysis data sets

In order to directly compare the transcriptome analysis of our AF-MSCs to another gestational tissue derived MSC populations, proven to be successfully utilized for the treatment of SARS-CoV-2-associated cytokine storm, and this showing a therapeutic potential, a meta-analysis was conducted to identify suitable UC-MSC RNA-seq datsets [50]. A comprehensive review of the existing literature was conducted to identify UC studies with associated publicly available datasets following PRISMA search terms and inclusion criteria/exclusion criteria. They included “Stem cells AND SARS-CoV-2” (996 outputs), “Stem cells AND SARS-CoV-2 AND cytokine storm” (167 outputs), “Umbilical cord stem cells AND SARS-CoV-2 AND cytokine storm” (12 outputs), and “RNA-Seq data available” (5 outputs). Initially 996 scientific articles were found in an NCBI “Stem cells AND SARS-CoV-2” search. After using the inclusion criteria and search terms shown above, 5 papers were selected due to their relatedness to the topic at hand and RNA-Seq data availability. The datasets from these publications were downloaded and analysed using the same informatics approaches to those employed for the AF-MSc RNA-seq.

*RNA-Seq dataset quality control*. Prior to in-depth analysis on the acquired data, a quality control check (FastQC) was carried out to identify any problems or points of concern. Since FastQC is calibrated for whole genome shotgun DNA sequencing rather than RNA-Seq, any “Fail” or “Warn” were investigated further in the context of the data being used. The variation in GC content transcript abundance were observed especially in “Per base sequence content” (Supplementary Fig. 2A) showing the percentage of each base in each position presented as four smooth lines around 25% and the low level of duplication in “Sequence Duplication Levels” (Supplementary Fig. 2B) indicated a high level of coverage of target sequence. FastQC also provides useful insight on RNA-Seq data by assessing whether the data is within a certain threshold for a specific parameter. For example, “Per base sequence quality” shows the good quality of values across all bases at each position in the FastQ file (Supplementary Fig. 2C). Additionally, phred scores of all AF-MSC samples were compiled to verify the quality of the base reads as shown in Table 3. With a mean phred score of 34.1, our AF-MSC samples exhibited a high quality of base call accuracy. Following the analysis of FastQc, all data was retained and mapped to the hg38 genome as a reference and mapping statistics reported according to ENCODE guidelines (Table 4).

**Table 3:** Phred scores of FastQC data replicates for the three samples of AF-MSCs

Samples	Q1%	Q10%	Q25%	Q50%	Q75%	Q100%	Mean
AF-MSCs #1	27	32	34	35	35	35	34.14404498
AF-MSCs #2	27	32	34	35	35	35	34.14888741
AF-MSCs #3	27	32	34	35	35	35	34.18170061

**Table 4:** Mapping statistics of amniotic fluid (AF) and umbilical cord (UC) mesenchymal stem cells (MSC) to the hg38 genome

Samples	Read length	Reads	Uniquely mapped (%)	Mapped to loci (%)	Unmapped (%)	Coverage
AF-MSC #1	75	31,525,592	92.81	6.43	0.76	1.33
AF-MSC #2	75	32,150,031	92.77	6.48	0.75	1.36
AF-MSC #3	75	32,782,263	92.84	6.42	0.74	1.38
UC-MSC #1	294	23,021,964	95.76	3.12	1.13	3.93
UC-MSC #2	294	26,478,121	95.30	3.04	1.66	4.5
UC-MSC #3	291	22,892,415	96.99	2.35	0.65	3.92
UC-MSC #4	293	22,561,500	96.99	2.21	0.80	3.89

### Differential expression of AF- and UC-MSCs

**Fig. 2:**
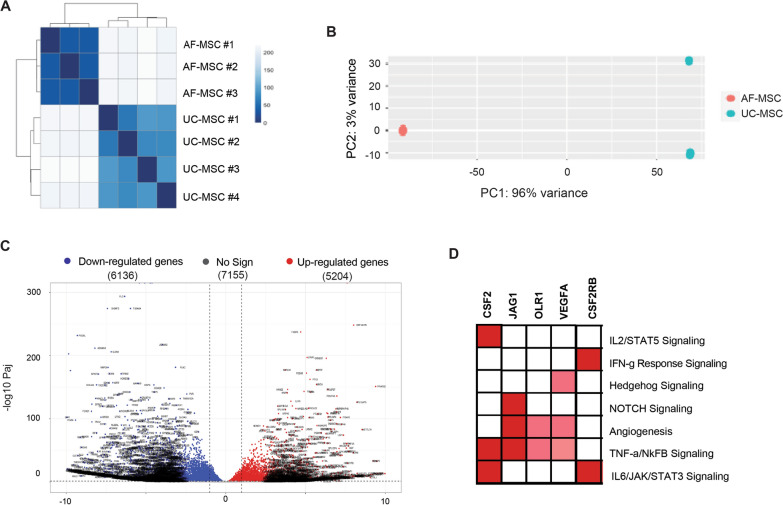
Transcriptome analysis reveals gene enrichment heatmaps between AF- and UC-MSCs. A correlation heatmap of Euclidean distance scores of normalized genes counts on all three amniotic fluid samples and all four umbilical cord samples **A**. Dendrograms show hierarchical clustering results, and the ColorBrewer Blues scale show the similarities between AF-MSC and UC-MSC samples. A principal component analysis plot of all three amniotic fluid samples and all four umbilical cord samples **B**. The PCA plot shows the uniqueness in the sample groups. Volcano plot displaying the all differentially expressed genes between AF- and UC-MSCs **C**. Volcano plot shows the magnitude of change (log fold change) versus *P* adjusted < 0.05. Red markers indicate significantly upregulated genes and blue markers indicate significantly down-regulated genes (< 0.05). Grey markers indicate genes where Padj > 0.05. Leading edge analysis heatmap on pathways of interest **D**. Red boxes represent highly enriched genes per the respective pathway. Pink boxes represent weaker yet present enrichment per respective pathway

DESeq2 was performed on all data sets and variance stabilizing transformation was applied to create a cluster heatmap and PCA plot, which are reported in Fig. 2 and provide an overview of the variance between the two sample types, AF-MSCs and UC-MSCs. A hierarchical clustering was applied to the heatmap to base the distance matrix on sample-to-sample distance, as shown via the dendrograms in the margins, rather than the physical distance between the rows/columns that comprise the matrix (Fig. 2A). Additionally, the sequential ColorBrewer Blues scale was used to differentiate between AF-MSC and UC-MSC samples. Importantly, no correlations between the two sample groups were identified, as indicated by the light blue/grey boxes in the lower left and upper right corners. The PCA plot reinforced the claim that both sample groups are unique (Fig. 2B). Significant differences exist between AF-MSC and UC-MSC samples with a percentage variance of 96%. Moreover, PC2 represents less significant secondary differences in the sample groups. These smaller differences were observed in one of the UC sample groups. After sample difference confirmation, a gross analysis of all the samples at gene level was performed. Genes were identified as differentially expressed when statistical significance between the two sample groups (AF- and UC-MSCs) was observed. To visualize the statistical significance (adjusted *p*-value; padj < 0.05 and base-2 log fold change > 2) a volcano plot (Fig. 2C) was produced with the latter focusing only on padj significance. Gene regulation difference is shown, with a base-2 log fold change > 2 on the sample groups. The dotted lines in the MA plot represent the threshold for base-2 log fold change of y = 1 and y = -1 (Supplementary Fig. 3). The red dots above the threshold of 1 depict up-regulated genes in UC-MSCs with at least a twofold expression compared to AF-MSCs. The blue dots below the threshold of base-2 log fold change of -1 represent downregulated genes in UC-MSCs. Genes selected for initial review in results Sect. "DEGs and pathway enrichment analysis" do not show a statistically significant differential expression. They include *Fn1*, *Sparc*, *Col1a1*, *Mtco1* and *Mt-rnr2*. Contrarily, *Col1a2* and *Col3a1* expressing statistically significant differential expression in UC-MSCs, show a lower expression compared to the level of differential expression was found for *Timp3* in AF-MSCs. Similar expression profile is consequently showed for AF- and UC-MSCs for these genes with *Col1a2* and *Col3a1* being more expressed in UC-MSCs and *Timp3* in AF-MSCs. Among the top ten most statistically significant differentially expressed genes across UC- and AF-MSCs, two genes are associated with UC-MSCs (*Mme* and *Eef1a1p5*), while the remaining eight are associated with AF-MSCs. Leading edge analysis was done to observe shared genes among pathways of interest, where all genes were related to generic immunomodulation or are specifically SARS-CoV-2 focused (Fig. 2D).

### DEGs and pathway enrichment analysis

**Fig. 3:**
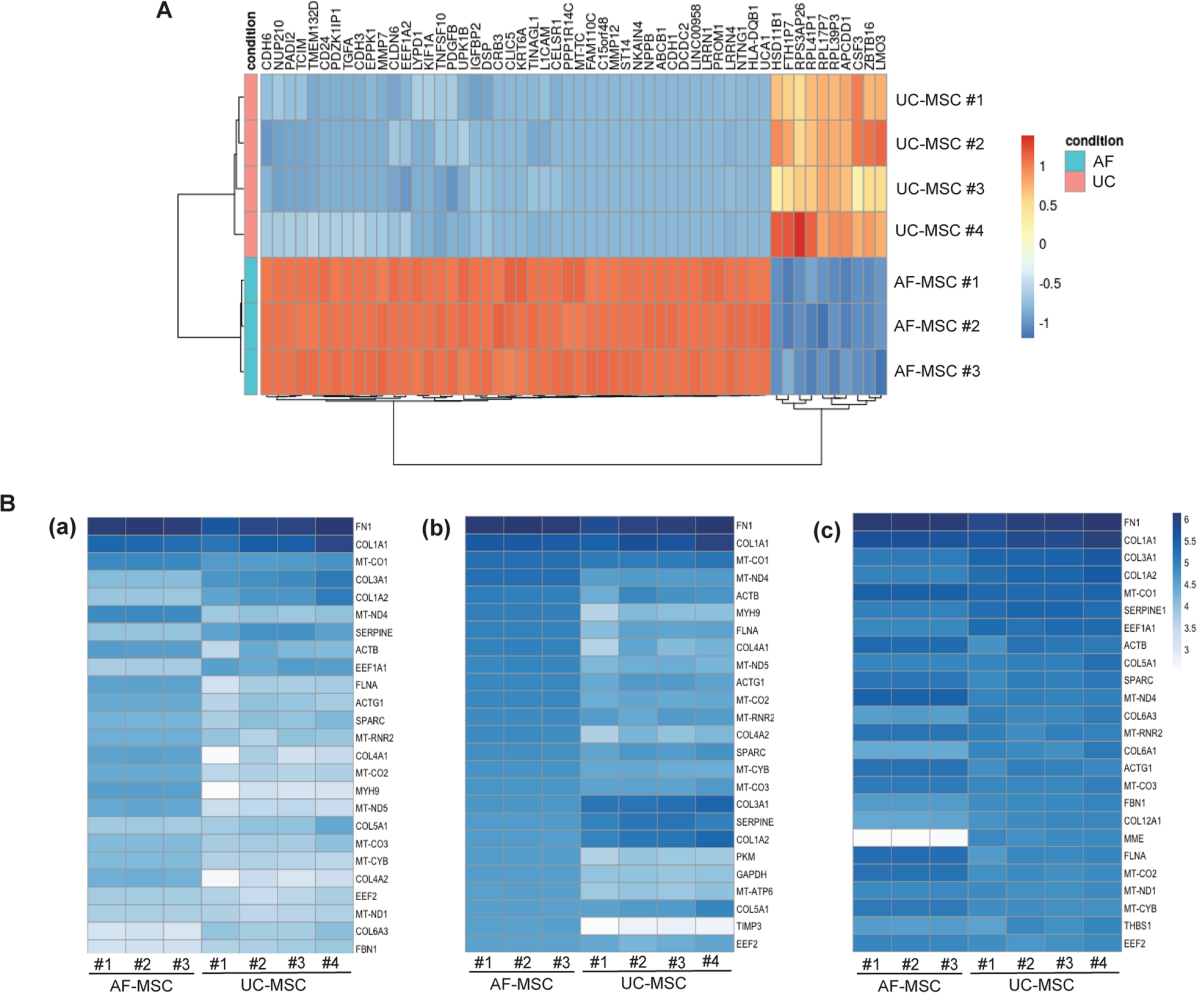
Evaluation of differentially expressed genes and the enrichment analysis. Hierarchical clustering heatmap showing top differentially expressed genes between both samples **A**. 25 highest expressed genes among the sample groups on three different count matrices **B**. The first heatmap **a** shows the top 25 highest expressed genes shared among AF-MSC and UC-MSC sample groups, the second heatmap **b** shows the highest expressed genes in AF-MSCs compared to UC-MSCs and the third heatmap **c** provides the highest gene expression in UC-MSCs and compares it to AF-MSCs. All differentially expressed genes have a *P* adjusted < 0.05

**Fig. 4:**
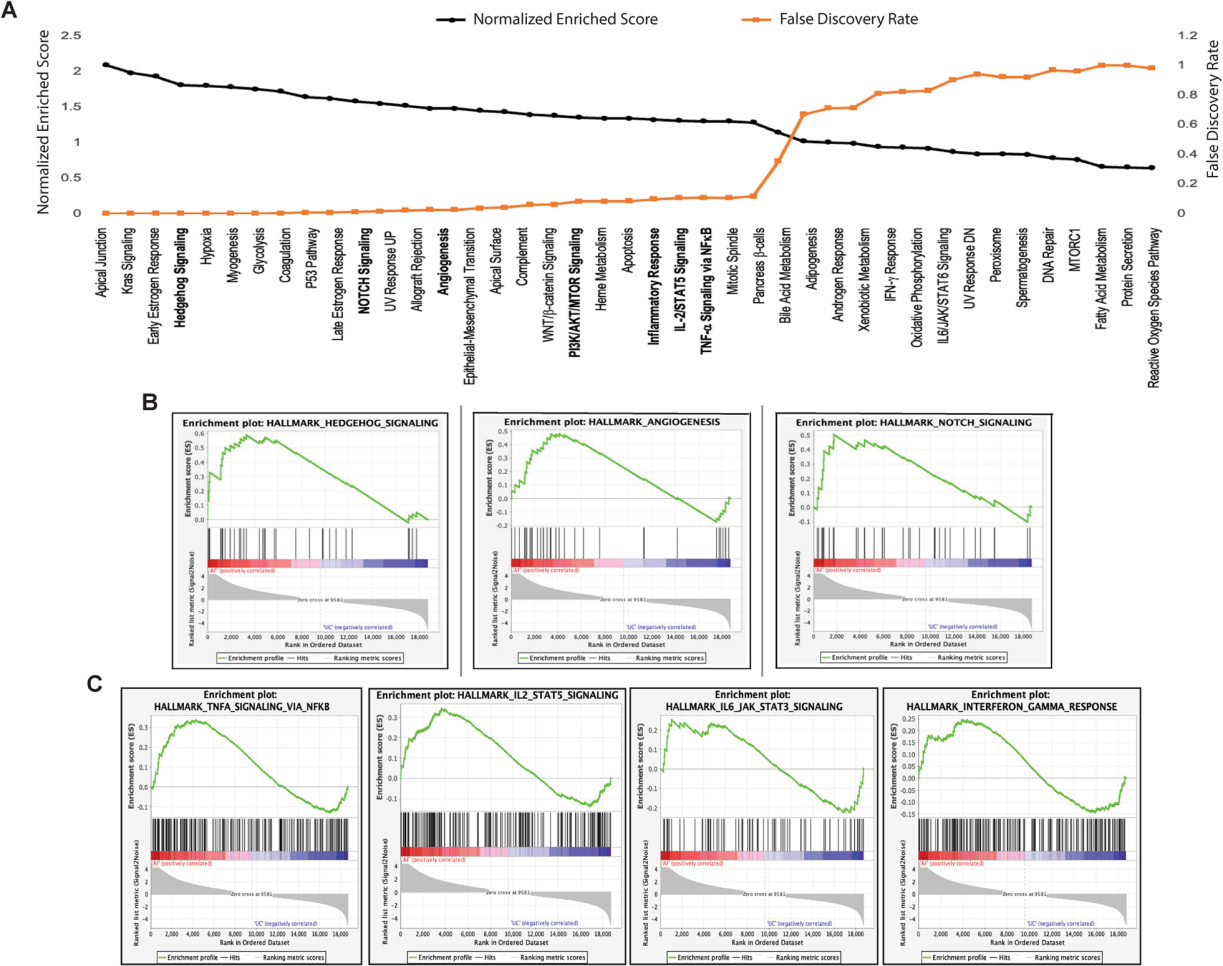
Gene set enrichment analysis of hallmark gene sets significantly enriched in AF- vs UC-MSCs. **A** Leading edge analysis showing enrichment scores (black line) and false discovery rates (orange line) of all 43 hallmarks pathways enriched in AF-MSCs. **B** MSCs related to immunomodulatory pathways enriched and their associated enrichment score plots for Hedgehog signaling pathway, Angiogenesis and NOTCH signaling. **C** SARS-CoV-2 associated cytokine storm pathways enriched and their associated enrichment score plots

Sequential heatmaps, derived from differential analysis data obtained from DESeq2, on three different count matrices, were created to visually observe the top 100 (Fig. 3A), showing the total differentially expressed genes between the UC- and AF-MSC samples, and then the 25 highest expressed genes among the sample groups (Fig. 3B a–c). For Fig. 4B, sequential heatmaps were chosen over divergent heatmaps due to their continuous scale and lack of a meaningful central value. The top 25 highest expressed genes shared among AF-MSC and UC-MSC sample groups are shown in Fig. 3B–a. The top five highest expressed genes shared between AF- and UC-MSCs include those responsible for encoding extracellular matrix proteins, including fibronectin (with *Fn1* being the most expressed gene) and collagen (with three genes being associated to type 1 and 3 collagen). The second heatmap shows the highest expressed genes in AF-MSCs compared to UC-MSCs (Fig. 3B–b). The genes *Fn1*, *Col1a1* and *Mt-co1* are observed to be the most highly upregulated in AF-MSCs. However, AF-MSCs express a unique gene signature as with genes involved in fundamental functions that dictate stem cell adhesion, differentiation, proliferation, and immune response. They include *Sparc*, *Mt-rnr2* and *Timp3*. The third heatmap provides the highest gene expression in UC-MSCs and compares it to AF-MSCs (Fig. 3B–c). The top two genes encode for extracellular matrix proteins, with some genes responsible to synthesize collagen. Specific signatures, however, also exist for UC-MSCs. They include the expression genes encoded for the protein translation machinery (*Eef1a*) and the mitochondrial respiratory chain (*Mt-co1*). In addition, a gene encoding for membrane metalloendopeptidase (*Mme*) and a member of the serine proteinase inhibitor (*Serpine*), were also found. In-depth analysis of DEGs was done with consideration to statistical significance, magnitude of comparison and mean expression levels. Incorporating these criteria allows for the possibilities of using thresholds based on cut-off points including *p*-value < 0.05 and log fold changes above one.

### Pathway enrichment analysis

Through utilization of powerful gene set enrichment tools such as GSEA and Leading-Edge analysis, individual gene level differences were analyzed for their gene pathway and functional differences. All genes were put through both forms of analysis and were mapped to the publicly available hallmarks data set found in the MSigDB collections. Pathways were shown in order of decreasing normalized enrichment score (NES) and visualized as black dotted line (Fig. 4A). However, pathways were ordered in NES decreasing order (from most enriched to least enriched) to consider the differences in gene set size and correlations among gene sets and the expression dataset directly taken from differentially analyzed RNA-Seq data. The significance threshold for False Discovery Rate (FDR) was set < 0.25 (25%). Out of the total 50 hallmark gene pathways, 43 of them were enriched in AF-MSCs. Within the 43 enriched pathways, 27 pathways possessed a false discovery rate of below 25%. These 27 pathways consist of the apical junction onwards until the mitotic spindle. Pathways following the mitotic spindle are considered statistically insignificant due to their nominal *p*-value exceeding 5% (0.05) and their false discovery rate surpassing 25%.

Pathways of particular interest include the NOTCH signaling, Hedgehog signaling, angiogenesis, IL2/STAT5 signaling, TNFα via NFKB, IL6/JAK/STAT3 signaling and IFN-g response. A visual depiction of the selected enriched stem cell related immunomodulation signaling, and SARS-CoV-2 associated cytokine pathways can be seen in the enrichment score plots shown in Fig. 4B–a and Fig. 4C, respectively. Contrary to AF-MSCs, UC-MSCs only represented negatively enriched pathways (Table 5).

**Table 5:** Hallmark gene sets negatively enriched pathways in UC-MSCs

Pathways	NES	FDR
Hallmark E2F Target	− 2.12	0.001
Hallmark MYC Target V1	− 1.92	0.001
Hallmark G2M Checkpoint	− 1.45	0.055
Hallmark Cholesterol Homeostasis	− 1.34	0.088
Hallmark Interferon-a Response	− 1.23	0.134
Hallmark MYC Target V2	− 1.11	0.256
Hallmark Unfolded Protein Response	− 1.04	0.369

## Discussion

COVID-19 pandemic waves and the long-term effects associated to its infection have shed light on the possibility to use MSCs as biological treatments to reduce inflammation and restore tissue homeostasis following SARS-CoV2 infection [51]. MSCs derived from fetal adnexa are promising tools for clinical applications, due their restorative, immunomodulatory and immunosuppressive features, which stem from tissues generally discarded at birth [52]. Relevantly, among gestational tissues, the therapeutic potential of the AF, amnion, and UC has been widely investigated for the treatment of several chronic and degenerative disorders, spanning from Crohn’s disease [53] to congenital malformations [54, 55]. However, the UC still represents the most used source of immunosuppressive MSCs, with the potential to counteract the chronic effects induced by COVID-19 infections and clinical trials have yet to observe the advantages of AF-MSCs over those isolated from other sources. In this study, we utilized NGS analysis to perform in-depth investigations into the hidden structure behind AF-MSCs to predict their application into potential therapeutics. Due to the demonstrated safety and therapeutic efficacy of UC-MSCs in clinical settings [28], these cells were used as a reference for the identification of AF-MSC-associated molecular moieties able to mitigate the chronic, inflammatory cascade associated to COVID-19 and restore a healthy, pro-regenerative environment. AF-MSCs were isolated during prenatal diagnosis and subjected to standard characterization before proceeding with the transcriptomic and bioinformatic analysis. Similarly to cells isolated from umbilical cord blood collected from premature neonate [56], quantitative PCR and flow cytometry demonstrated the presence of a homogeneous population of highly proliferative cells, expressing canonical mesenchymal markers (*Cd44, Cd29, Cd90, Cd73* and *Cd105*) but also pluripotent (*Nanog* and *Oct4*) genes, supporting their undifferentiated phenotype and intermediate state between adult and embryonic stem cells [57, 58]. Cells isolated during the first gestation period display an undifferentiated phenotype and these primitive properties could be related to the higher concentration of stem/progenitor cells within the fetal and adnexal fetal tissues, as well as to a more rapid progression in cell cycle required for fetuses’ development to drive an intense organogenesis [56]. According to previous studies reported by us and others, however, a decay in their proliferation and doubling time was observed overtime (from P1 to P5) in culture, suggesting their use MSCs within passage 5 for clinical applications [59, 60]. For this reason, subsequent studies were performed using cells at P3. When the transcriptomic profile of AF- and UC-MSCs was compared, bioinformatic analysis revealed stark differences at the sample level with marginal inter-sample variability. This discrepancy was expected as samples were handled differently and at different laboratories. Similar gene expression profiles were identified between AF- and UC-MSCs. Markedly, among the top five expressed genes in both cell lines, three of them provide instruction for collagen production. Type 1 collagen, encoded by *Col1a1* and *Col1a2*, alongside type 3 collagen, encoded by *Col3a1*, are essential in embryogenesis as they play key roles in the formation of bone, skin, tendons, cornea, blood vessels and brain development. They are heavily involved in the formation and remodeling of tissues’ extracellular matrix and are therefore crucial to achieve the complex task of wound healing [61]. Higher levels of genes encoding for collagen (*Col1a1* and *Col1a2*) were found in UC-MSCs compared to AF-MSCs. Similar to UC-MSCs, genes *Fn1*, *Col1a1* and *Mt-co1* are observed to be the most highly upregulated ones in AF-MSCs. *Fn1*, the top expressed gene in both groups, is responsible for encoding fibronectin, a multifunctional adhesive glycoprotein directly involved in embryogenesis due to its active participation in tissue repair and regulation of cell attachment and motility [62]. Enriched genes in AF-MSCs also include *Sparc*, *Mt-rnr2* and *Timp3*, which are involved in immunoregulation. Specifically, *Sparc* regulates cellular properties via interactions with cytokines and extracellular matrix [63], thus dictating stem cell adhesion, differentiation, proliferation and immune response [64], *Mt-rnr2* suppresses apoptosis and reduces fibrosis, and *Timp3* is a protein coding gene tissue inhibitor of matrix metalloproteinases that control immune related processes such as cell growth/death, angiogenesis (VEGF, EGF) and inflammation (TNF) [65, 66]. In AF-MSCs *Timp3* is expressed at a noticeably higher rate than UC-MSCs, suggesting its potential role as a negative regulator of excessive cytokine production [67].

AF-MSCs also display a unique gene signature. The MA plot reported in Supplementary Fig. 3 shows the top ten most statistically significant differentially expressed genes across AF- and UC-MSCs. Out of them, only two are associated to UC-MSCs and the remaining eight genes are associated to AF-MSCs. They include *Pmepa1* and *Sh3rf2* that play key roles in immunomodulation. *Pmepa1* regulates the TGF-β signaling thus making it a key contributor to cell proliferation and differentiation, apoptosis, motility, extracellular matrix production and immunosuppression [68] suggesting a potential role for AF-MSCs in turning off the TGF-β pathway activated by the cyclical pro-inflammatory environment induced by SARS-CoV-2 infection [69]. Furthermore, *Sh3rf2* is an anti-apoptotic regulator of the c-Jun N-terminal kinases (JNK) pathway which is responsible for controlling cellular responses towards detrimental extracellular forces, specifically pro-inflammatory cytokines [70]. When the JNK pathway is activated by pro-inflammatory cytokines a signaling cascade starts inducing a detrimental apoptotic effect. AF-MSCs can modulate this response with *Sh3rf2* shutting down the JNK pathway through anti-apoptotic regulation. The fact that these two genes are differentially expressed in AF-MSCs and not in UC-MSCs suggests that the former may offer an advantage in attenuating the cytokine storm. We confirmed the immunosuppressive role of AF-MSCs preconditioning them with a pro-inflammatory cocktail consisting of cytokines identified as key mediators to the MSCs activation [71] (TNF-α and IFN-γ, 20 ng/ml) secreted immunosuppressive and immunomodulatory factors (*Pge-2*, *Tnf-α*, and *Tgf- b*) [72]. In particular, the *Tgf-b* pathway is known to orchestrate several mechanisms within MSCs, including self-renewal, differentiation [73] and their potential to modulate macrophage behavior skewing their phenotype from pro-inflammatory to anti-inflammatory [74].

A functional experiment was set up to test the effectiveness of these observations and AF-MSCs were exposed to activated monocytes by means of different strategies: they were cultured in direct contact with immune cells or physically separated through a transwell system. To determine whether the immunosuppressive effect is mediated by paracrine factors, an additional group was included, consisting of monocytic cells only exposed to the media conditioned by AF-MSCs. In all cases, a drastic reduction in the proliferation of activated monocytes was observed, confirming the immunosuppressive potential of AF-MSCs [75] for our lines established from patient samples. Our data demonstrate that direct contact between AF-MSCs and ThP-1 cells allows for such a decrease [76] although soluble factors can achieve a similar immunosuppressive effect [77–79] and attenuating their functionality [80, 81]. A similar trend has been widely documented for UC-MSCs by various research groups globally. Their therapeutic effect has been shown to be mediated by the secretion of soluble factors, including PGE2, TGF-β, and IL-6 [82], which are effective in reducing lymphocyte proliferation [83, 84] and modulating macrophages toward an anti-inflammatory phenotype. Evidence supports their efficacy in attenuating lung injury [85], improving pancreatic function in type 2 diabetic mice [86], and reducing rheumatoid arthritis symptoms [87] in preclinical studies. In this study, we also explored the immunomodulatory role of patient-derived AF-MSCs alongside their immunosuppressive potential. Our data demonstrate that exposure of undifferentiated macrophages (MΦ) to media conditioned by AF-MSCs, either cultured under standard conditions or stimulated with inflammatory cytokines (TNF-α and IFN-γ, iAF-MSCs), led to an upregulation of markers associated with the MΦ2 phenotype, along with a reduction in levels of pro-inflammatory factors commonly recognized as immunomodulatory. Contrary to existing literature [88–90], in this study, the highest expression levels of anti-inflammatory markers were observed when MΦs were exposed to media conditioned by non-inflamed AF-MSCs, rather than inflamed ones. Interestingly, inflamed macrophages (MΦ1) exhibited a decrease in the expression of pro-inflammatory markers (iNOS, IL-6, and TNF-α) after exposure to the same conditioned media, with iAF-MSCs eliciting the most pronounced effect.

Accumulating a total of 43 enriched hallmark pathways compared to the 7 found in UC-MSCs (Table 5), AF-MSCs display a broader immunomodulatory potential. Not surprisingly, biological pathways enriched in AF-MSCs include the Hedgehog and NOTCH signaling pathway, both involved in embryo development [91, 92], tissue homeostasis and repair [93, 94]. This evidence confirms the role amniotic fluid components play as mediators of the communication required to support correct embryogenesis. Recently, however, the activation of the Hedgehog and NOTCH signaling pathways following viruses’ infection (including SARS-CoV-2) has been reported to induce STAT3 upregulation, to increase IL-6 expression, and lead to the observed detrimental outcomes, such as fibrosis [95]. It is conceivable that the potential of AF-MSCs to regulate these molecular mechanisms will pave the way for the development of effective and broad-spectrum inhibitors of the pathogenic responses associated to SARS-CoV-2. Our analysis also identified other immunomodulatory pathways, usually enriched in UC-MSCs [96–98], as highly enriched in AF-MSCs. Among them, IL-2/STAT, TNF-a/NFkB, IL-2/STAT5, PI3K/AKT/MTOR signaling pathways have been reported to be actively involved in the SARS-CoV-2-cytokine storm [3]. This data is substantiated by experimental evidence on the role AF-MSCs play in the differentiation of CD4 + T cells (known to otherwise release IL-2 and induce a cyclical pro-inflammatory environment [3]) into cell subsets that co-express high levels of molecules (i.e., CD25 and FoxP3). These molecules are generally suppressed by SARS-CoV-2 infection [99, 100] and their production may result in an effective counteracting of the chronic effect induced by cytokine storm. A similar trend has been observed in the TNFα-mediated NF-κB pathway, which is thought to contribute significantly to the hyperactivation of the SARS-CoV-2-associated cytokine storm by inducing epithelial cell apoptosis [51]. The regulation of this pathway is significantly enriched in AF-MSCs and exposure to AF-MSCs (and their derivatives) has the potential to attenuate the cytokine storm more efficiently than UC-MSCs due to the higher enrichment among all major applicable pathways. Another pathway likely to be targeted by AF-MSCs is the PI3K/AKT signalling pathway, mTOR and nuclear factor kappa B, which is mostly linked to the pathogenicity of SARS-CoV-2 [101]. Inhibition of mTOR followed by the PI3K/AKT signalling pathway suppression has been suggested to impair the SARS-CoV-2 detrimental effects in different studies [102, 103].

Altogether, the functional and transcriptomic data presented in this study provide a comprehensive overview of the potential role that -MSCs may play in mitigating the life-threatening effects of cytokine storms. While this study specifically focuses on the ability of AF-MSCs to reduce the cytokine storm associated with SARS-CoV-2 infection, these findings suggest that AF-MSCs hold broader therapeutic potential for managing cytokine storms induced by a variety of other conditions [104]. Our dataset supports the notion that AF-MSCs modulate key biological pathways that are common molecular signatures of pathogen-induced inflammation, regardless of the infective agent. These pathways include those triggered by bacterial infections such as Group A Streptococcus (GAS), *Staphylococcus aureus*, *Francisella tularensis*, *Klebsiella pneumoniae*, and *Yersinia pestis*), as well as viral infections like influenza A virus (IAV), Ebola virus, and coronaviruses (SARS-CoV, MERS-CoV), which predominantly engage the TNF and JNK signaling pathways [105]. Furthermore, AF-MSCs also target inflammatory pathways associated with autoinflammatory disorders or monogenic inflammation, including PI3K/AKT, mTOR, and NF-κB signaling ([106–108]. Notably, these pathways are implicated in a range of pathophysiological processes, including therapeutic interventions inducive of cytokine storm (i.e., CAR-T therapy [109]). In such contexts, the PI3K/AKT/mTOR signaling axis is often activated, underscoring the potential of AF-MSCs to regulate inflammation in diverse clinical scenarios. Taken together, these findings suggest that AF-MSCs could serve as a versatile therapeutic strategy for managing various forms of inflammation, both infectious and non-infectious, by modulating critical inflammatory pathways that are central to disease progression [110].

MSCs have garnered significant attention for their therapeutic potential across various medical fields, with a growing body of evidence supporting their safety in clinical applications. While they offer numerous therapeutic advantages over their adult counterparts, it is essential to recognize some potential risks, including the risk of contamination during cell isolation and prolonged in vitro expansion. Despite strict adherence to protocols, the accumulation of genetic aberrations remains a concern. Culture-expanded MSCs may exhibit significant alterations in cell morphology, physiology, and function, contributing to increased heterogeneity [111, 112], and spontaneous malignant transformation has been reported in approximately 46% of human MSCs after four weeks of culture [111]. However, it is critical to note that these risks are not uniform across all MSC sources as MSCs from chorionic villi, UC and AF have been shown to possess greater stability. They do not exhibit chromosomal aberrations in vitro, even up to the 15th passage [113]. Clinical trials utilizing UC-MSC-based therapy for the treatment of patients with coronavirus disease since 2019 have shown promising long-term efficacy, with UC-MSCs significantly improving whole-lung lesion volume, reducing mortality rates (notably increasing survival by about 2.5-fold), and preventing long-term pulmonary disability [114]. However, it is important to note that, although less frequently reported, adverse effects such as thromboembolism following hUC-MSC infusion in kidney transplant patients and glomerular or tubular damage due to overdosing of cell therapeutics have been observed [115]. Nevertheless, based on our data, AF-MSCs offer distinct advantages over their umbilical cord counterparts, potentially providing a safer and more effective alternative for clinical applications. A promising strategy to overcome potential limitations associated with MSC-based therapies while harnessing their therapeutic properties is the use of MSC-derived extracellular vesicles (EVs). Unlike MSCs, EVs do not carry the risk of uncontrolled proliferation, tumorigenicity, or genetic aberrations since they are acellular [116]. Moreover, MSC-derived EVs retain many of the beneficial properties of MSCs, including their ability to modulate immune responses, promote tissue regeneration, and carry therapeutic molecules such as proteins, lipids, and microRNAs [116]. Given the numerous beneficial properties of MSC-derived EVs, they represent a promising alternative to MSCs themselves [40]. However, further research is necessary to fully understand EVs, particularly due to the complexity and heterogeneity of their cargo, which may influence multiple cellular pathways besides the intended mechanism of action. A key area of ongoing investigation is whether MSCs used for EV production require priming prior to EV extraction, a question raised by several studies proving that MSCs exposure to pro-inflammatory environments determines alterations in EV biogenesis pathways, affecting their cargo and, ultimately, their therapeutic outcome [115, 117].

## Conclusion

Despite significant progress since the emergence of this disease, the cytokine storm triggered by COVID-19 infections highlights the urgent need for a safe, effective, and broadly applicable therapeutic strategy. Indeed, the long-term lung damage observed in certain patient subgroups demands immediate attention. Our direct comparison of transcriptomic profiles from clinically relevant UC-MSCs showcases the distinct advantages of AF-MSCs, particularly their enhanced immunosuppressive properties. These findings align with existing literature supporting the potential of AF-MSCs to modulate inflammation, restore microenvironmental balance, and protect pulmonary epithelium following alveolar injury, as demonstrated in various preclinical models. The data presented here not only reinforce the therapeutic promise of AF-MSCs in managing cytokine storms but also offer a strong rationale for their potential in treating other life-threatening inflammatory conditions. Given their ability to target critical inflammatory pathways shared across various infectious and non-infectious diseases, AF-MSCs represent a compelling candidate for future clinical studies aimed at evaluating their efficacy in controlling excessive inflammation, particularly in the context of SARS-CoV-2 and other severe inflammatory responses. Moving forward, a comparative analysis between patient-derived AF-MSCs and UC-MSCs, including the molecular profiles of EVs isolated from them, would offer a comprehensive understanding of their immunomodulatory roles, ensuring that all variables and conditions are thoroughly addressed.

## Supplementary Information

Supplementary file 1.

Supplementary file 2.

Supplementary file 3.

## Data Availability

Raw and processed datasets have been deposited in NCBI’s Gene Expression Omnibus (GEO) (https://www.ncbi.nlm.nih.gov/geo) with accession reference GSE240855.

## Abbreviations

AF-MSCs: Amniotic fluid-derived MSCs
AF: Amniotic fluid
AKT: Protein kinase B
APC: Allophycocyanin
CFU-F: Colony-forming unit-fibroblastic
CM: Conditioned media
CRS: Cytokine release syndrome
CVD: Cardiovascular disease
DEG: Differentially expressed gene
DT: Doubling time
FBS: Fetal bovine serum
FDR: False discovery rate
FITC: Fluorescein isothiocyanate
GEO: Gene expression omnibus
GSEA: Gene set enrichment analysis
HG-DMEM: High glucose-dulbecco’s modified eagle medium
IFN-γ: Interferon-gamma
IL: Interleukin
JNK: Jun N-terminal kinases
MSCs: Mesenchymal stem cells
mTOR: Mammalian target of rapamycin
NCBI: National center for biotechnology information
NES: Normalized enrichment score
NGS: Next generation sequencing
P/S: Penicillin/streptomycin
P2: Passage 2
PCA: Principal component analysis
PE: Phycoerythrin
PFA: Paraformaldehyde
PGE2: Prostaglandin E2
PHA: Phytohemagglutinin
PI3K: Phosphoinositide 3-kinases
QC: Quality control
RPMI: Roswell park memorial institute
RT: Room temperature
Sars-Cov-2: Severe acute respiratory syndrome coronavirus 2
SD: Standard deviation
SRA: Sequence reads archive
STAT: Signal transducer and activator of transcription factor
TGF-b: And tumor growth factor-beta
Th2 cells: Influence pro-inflammatory T-helper 2 cells
TNF-α: Tumor necrosis factor-alpha
UC: Umbilical cord
UC-MSCs: Umbilical cord-derived MSCs
