# Supplementary figures and images for: Amniotic fluid-derived mesenchymal stem cells as a therapeutic tool against cytokine storm: a comparison with umbilical cord counterparts
Source: Stem Cell Res Ther. 2025 Mar 28;16:151. doi: 10.1186/s13287-025-04262-0 (PMC11951844; doi:10.1186/s13287-025-04262-0)

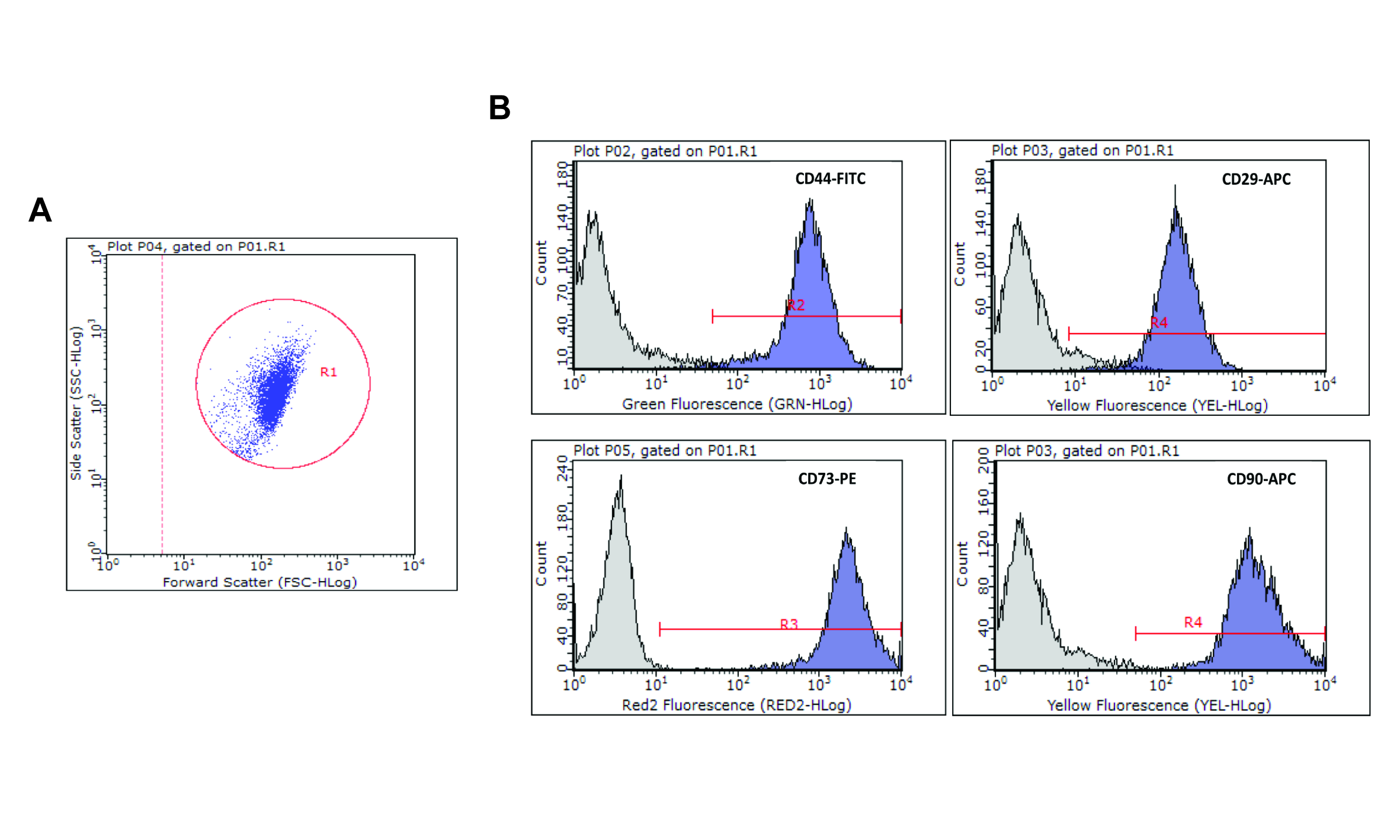

Supplement: Supplementary file 1 [file 13287_2025_4262_MOESM1_ESM.tif]

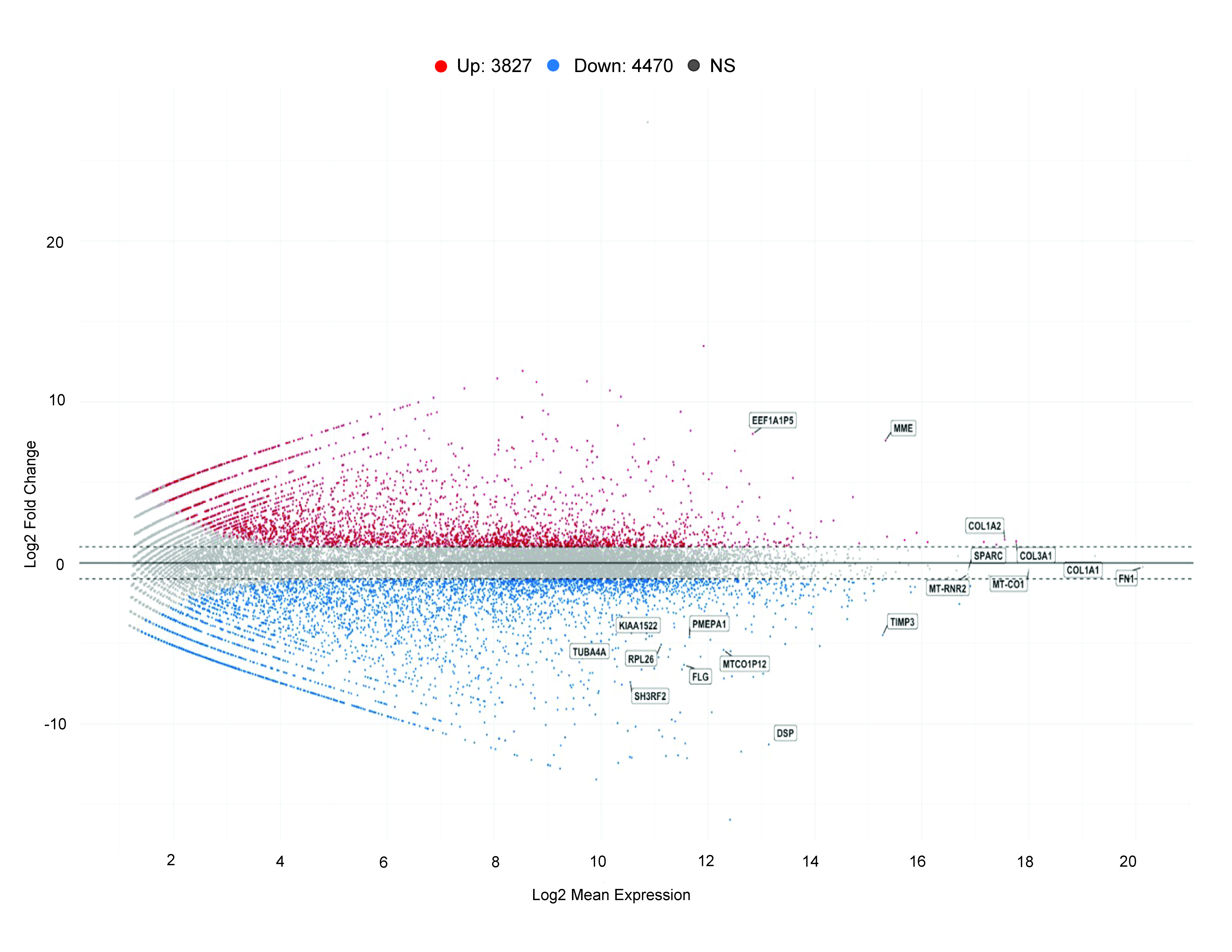

Supplement: Supplementary file 3 [file 13287_2025_4262_MOESM3_ESM.tif]
